# Second allogeneic transplants for multiple myeloma: a report from the EBMT Chronic Malignancies Working Party

**DOI:** 10.1038/s41409-021-01286-x

**Published:** 2021-05-11

**Authors:** Patrick J. Hayden, Dirk-Jan Eikema, Liesbeth C. de Wreede, Linda Koster, Nicolaus Kröger, Hermann Einsele, Monique Minnema, Alida Dominietto, Michael Potter, Jacob Passweg, Arancha Bermúdez, Stephanie Nguyen-quoc, Uwe Platzbecker, Johanna Tischer, Fabio Ciceri, Joan Hendrik Veelken, Per Ljungman, Nicolaas Schaap, Edouard Forcade, Angelo Michele Carella, Virginie Gandemer, William Arcese, Adrian Bloor, Attilio Olivieri, Laure Vincent, Meral Beksac, Stefan Schönland, Ibrahim Yakoub-Agha

**Affiliations:** 1 Department of Haematology, Trinity College Dublin, St. James’s Hospital, Dublin, Ireland; 2 EBMT Statistical Unit, Leiden, The Netherlands; 3 Department of Biomedical Data Sciences, LUMC Leiden, The Netherlands; 4 EBMT Data Office Leiden, Leiden, The Netherlands; 5 University Hospital Eppendorf, Hamburg, Germany; 6 Universitätsklinikum Würzburg, Würzburg, Germany; 7 University Medical Center Utrecht, Utrecht, The Netherlands; 8 IRCCS Ospedale Policlinico San Martino, Genova, Italy; 9 Royal Marsden Hospital, London, UK; 10 University Hospital, Basel, Switzerland; 11 Hospital Universitario Marqués de Valdecilla, Santander, Spain; 12 Hopital la Pitié-Salpêtrière, Paris, France; 13 Medical Clinic and Policinic 1, Leipzig, Germany; 14 Klinikum Grosshadern, Munich, Germany; 15 Ospedale San Raffaele s.r.l., Milano, Italy; 16 Leiden University Hospital, Leiden, The Netherlands; 17 Karolinska University Hospital, Stockholm, Sweden; 18 Radboud University Medical Centre, Nijmegen, The Netherlands; 19 CHU Bordeaux, Pessac, France; 20 Casa Sollievo della Sofferenza, San Giovanni, Italy; 21 Centre Hospitalier Universitaire de Rennes Hôpital sud, Rennes, France; 22 Rome Transplant Network ¨Tor Vergata¨ University of Rome, Rome, Italy; 23 Christie NHS Trust Hospital, Manchester, UK; 24 Azienda Ospedali Riuniti di Ancona, Ancona, Italy; 25 CHU Lapeyronie, Montpellier, France; 26 Ankara University Faculty of Medicine, Ankara, Turkey; 27 Medical Department V, University Hospital Heidelberg, Heidelberg, Germany; 28 Univ. Lille, CHU Lille, INSERM, Infinite, U1286, Lille, France

**Keywords:** Stem-cell therapies, Immunotherapy

## Abstract

The EBMT Chronic Malignancies Working Party performed a retrospective analysis of 215 patients who underwent a second allo-HCT for myeloma between 1994 and 2017, 159 for relapse and 56 for graft failure. In the relapse group, overall survival (OS) was 38% (30–46%) at 2 years and 25% (17–32%) at 5 years. Patients who had a HLA-identical sibling (HLAid-Sib) donor for their first and second transplants had superior OS (5 year OS: HLAid-Sib/HLAid-Sib: 35% (24–46%); Others 9% (0–17%), *p* < 0.001). There was a significantly higher incidence of acute grade II-IV GvHD in those patients who had also developed GvHD following their initial HLA-identical sibling allo-HCT (HLAid-Sib/HLAid-Sib: 50% (33–67%); Other 22% (8–36%), *p* = 0.03). More as opposed to fewer than 2 years between transplants was associated with superior 5-yr OS (31% (21–40%) vs. 10% (1–20%), *P* = 0.005). On multivariate analysis, consecutive HLA-identical sibling donor transplants conferred a significant OS advantage (0.4 (0.24–0.67), *p* < 0.001). In the graft failure group, OS was 41% at 2 years. In summary, a second allo-HCT using a HLA-identical sibling donor, if available, provides the best transplant outcomes for relapsed myeloma in this setting.

## Introduction

Although allogeneic haematopoietic cell transplantation (allo-HCT) is not routinely performed in patients with multiple myeloma (MM), this approach is still offered to patients with high-risk disease. According to the EBMT database, there were 2684 MM patients transplanted in EBMT centres between 2013 and 2018. Post-transplant relapse remains the main cause of treatment failure. Options for the treatment of disease relapse following allo-HCT include salvage chemotherapy, novel targeted agents, and, increasingly, immunotherapies [1–4]. In selected patients who achieve clinical remission, there may be a role for a second allo-HCT [5–10].

The first reports of outcomes following second allogeneic transplants to treat disease relapse were characterised by prohibitively high rates of non-relapse mortality (NRM) of up to 45% [11, 12]. Less toxicity was seen with the use of reduced intensity conditioning (RIC) regimens. Shaw and colleagues performed a retrospective U.K. multi-centre analysis of 71 patients receiving a second allogeneic transplant using RIC after disease relapse following an initial myeloablative allo-HCT [5]. The predicted overall survival (OS) and NRM at 2 years were 28% and 27%, respectively. In a subsequent EBMT registry study of 234 adult patients with acute leukaemia who received a second RIC transplant between 2000 and 2012 as salvage treatment for relapse following an initial RIC allo-HCT, the cumulative NRM and OS rates at 2 years were 22.4%, and 20.5%, respectively [6].

In a 2015 retrospective EBMT study of 2632 second allogeneic transplants performed to treat disease relapse following a first transplant, the factors associated with better survival included the use of a HLA-identical sibling donor for the second transplant, low disease burden, longer remission duration after the first transplant, a longer interval between the transplants, younger age, the absence of grade II–IV acute graft-versus-host disease (GvHD) or chronic GvHD after the first transplant, and later year of transplant [13]. There was no difference in overall survival (OS) between those transplanted using their original donors when compared to those who were transplanted using new donors.

Second allogeneic transplants have also been performed to treat graft failure. Ferra and colleagues reported a 5 year OS of 31% in eighty patients who underwent a second transplant for graft failure [14]. Neutropenic patients and those transplanted using a second unrelated donor fared less well [15, 16].

There has, to date, only been one small report specifically relating to second allogeneic transplants in patients with myeloma following either disease relapse or graft failure [17]. We, therefore, performed a retrospective analysis of 215 patients with myeloma who underwent second allogeneic transplants between 1994 and 2017.

## Methods

### Study design

This study was performed in accordance with the principles of the Declaration of Helsinki and was approved by the EBMT, a nonprofit, scientific society representing more than 600 transplant centres, mainly in Europe. All data is stored in a central database. Patients’ informed consent was obtained locally according to regulations. The study cohort consisted of 215 patients with myeloma who were reported to the EBMT registry as having undergone a second allo-HCT either for relapsed myeloma or for graft failure. Second transplants performed for donor-derived haematological malignancies were excluded. The data were obtained from the EBMT data registry.

Available donor information is categorized as either HLA-identical sibling (HLAid-Sib) or other, the latter group being composed of matched related and unrelated and mismatched related and unrelated donors.

Disease stage was classified using the EBMT registry nomenclature regarding remission status at HSCT. Patients were classified as having either Low (PR or better) or Advanced (SD, MR, relapse, progression, primary refractory/no CR) stage disease, in this case, myeloma. In other words, patients with myeloma in less than a partial remission pre-transplant were considered to have advanced stage disease.

The primary outcomes of interest were OS, non-relapse mortality (NRM), relapse/progression, relapse/progression-free survival (PFS), and causes of death. Outcomes are provided at 2 or 5 years after the second HCT, depending on the availability of a sufficient number of patients in each of the subgroups of interest. OS and PFS were estimated using the Kaplan–Meier product limit estimation method, and differences in subgroups were assessed by the Log-Rank test. Median follow-up was determined using the reverse Kaplan–Meier method.

The cumulative incidence of relapse and NRM were analysed together in a competing risks framework. Neutrophil engraftment was defined as an absolute neutrophil count ≥0.5 × 10^9^/L for three consecutive days. The cumulative incidence of neutrophil engraftment is provided at day 28 after the second transplant, with the competing event being death without neutrophil engraftment. Competing risks analyses were also applied to estimate the incidences of acute grade II-IV GvHD and limited and extensive chronic GvHD (cGvHD), by day 100 and 2 and 5 years, respectively. Subgroup differences in cumulative incidences were assessed using Gray’s test.

Multivariable Cox regression was applied to investigate the simultaneous impact of multiple covariates on outcomes, when a sufficient number of patients and subsequent events were available. For OS and PFS, hazard ratios are provided, whereas for the competing risks outcome relapse, cause-specific hazard ratios are provided for the events of interest, both denoted as HR. Included covariates were used in all outcomes: Patient age at second transplant (in decades), patient sex, donor type (HLA identical sibling at both first and second transplant versus other), interval between first and second transplant (years), disease risk (low versus advanced), conditioning intensity (reduced versus myeloablative) and any previous GvHD (no versus yes). All models are stratified by categorized year of second transplant (≤2008, >2008).

Continuous variables are presented in text as median (range) and categorical variables as percentages within the group of patients with available data. All estimates are reported with corresponding 95% confidence intervals in parentheses. All *p* values were two-sided and *p* < 0.05 was considered significant. Statistical analyses were performed in R version 3.6.0 (R Development Core Team, Vienna, Austria), using packages ‘survival’, ‘prodlim’ and ‘cmprsk’.

## Results

Patient characteristics in the relapse (*n* = 159) and graft failure (*n* = 56) cohorts are shown in Table 1A.

**Table 1:** (A) Patient characteristics at first and second transplants. (B) Number of transplants in each 5-year period for relapsed and graft failure patients. (C) Rates of acute and chronic GVHD and of engraftment following the second transplant.

A												
	Relapse cohort (*n* = 159)						Graft failure cohort (*n* = 56)					
	1st allo-HCT			2nd allo-HCT			1st allo-HCT			2nd allo-HCT		
	Missing	*N*	%	Missing	*N*	%	Missing	*N*	%	Missing	*N*	%
Gender												
M		60	37.7				24	42.9				
F		99	62.3				32	57.1				
MM												
IgA	8	33	21.9				13	23.2				
IgG		77	51				25	44.6				
LC		36	3.8				18	32.1				
Non-sec		3	2									
Other		2	1.3									
Graft												
BM	2	47	29.9	2	15	9.6	1	12	21.8	1	6	10.9
CB		1	0.6		1	0.6		2	3.6			
PB		109	69.4		141	89.8		41	74.5		49	89.1
Donor												
HLA Id Sib	2	108	68.8	3	95	60.9		23	41.8	1	21	38.2
Other		49	31.2		61	39.1		32	58.2		34	61.8
Change of donor												
Other				57	58	56.9				16	17	42.5
Same					44	43.1					23	57.5
EBMT score												
<4	11	49	33.1	15	3	2.1	3	7	13.2	8	2	4.2
4		40	27		18	12.5		12	22.6		9	18.8
5		39	26.4		72	50		20	37.7		22	45.8
>5		20	13.5		51	35.4		14	26.4		15	31.2
Conditioning												
Reduced intensity	17	84	59.2	26	73	54.9	3	42	79.2	8	33	68.8
Myeloablative		58	40.8		60	45.1		11	20.8		15	31.2
Karnofsky												
<80	40	7	5.9	33	25	19.8	11	1	2.2	11	17	37.8
80–100		112	94.1		101	80.2		44	97.8		28	62.2
Stage												
Low	10	116	77.9		60	41.4	2	46	85.2	8	29	60.4
Advanced		33	22.1		85	58.6		8	14.8		19	39.6
Interval Tx1–Tx2 (months)					159	40.5 (1.5–170.4)					56	3.5 (0.7–66.6)
Age at Tx1 (yrs.) Med (range)			48 (20–63)						49 (25–68)			
Year of Tx1 Med (range)			2004 (1985–2016)						2008 (1996–2017)			
Age at Tx2 (yrs.) Med (range)						51 (29–69)				56		50 (25–68)
Year of Tx2 Med (range)						2008 (1994–2017)				56		2008 (1996–2017)

B				
	Relapsed		Graft failure	
Period	*N*	%	*N*	%
1994–1998	14	8.8	2	3.6
1999–2003	26	16.4	10	17.9
2004–2008	41	25.8	16	28.6
2009–2013	43	27.0	19	33.9
2014–2017	35	22.0	9	16.1
Total	159	100.0	56	100.0

C					
	Donor	Relapsed myeloma (*n* = 159)	*p* value	Graft failure (*n* = 56)	
GVHD					
Acute grade II GVHD		16% (10–22%)		12% (3–22%)	
Acute grade III–IV GVHD		14% (8–19%)		15% (5–25%)	
Death without GVHD		12% (7–18%)		17% (6–27%)	
Acute grade II–IV GVHD	Other	25% (14–36%)	0.2	29% (13–45%)	0.865
	HLAid-Sib-HLAid-Sib	34% (23–45%)		25% (4–46%)	
Death without acute GvHD	Other	14% (6–23%)	0.619	19% (5–33%)	0.539
	HLAid-Sib-HLAid-Sib	11% (4–18%)		12% (0–29%)	
Acute GVHD with first allo	Other	22% (8–36%)	**0.03**		
Acute GVHD with first allo	HLAid-Sib-HLAid-Sib	50% (33–67%)			
No acute GVHD with first allo	Other	26% (8–44%)		17% (0–34%)	0.53
No acute GVHD with first allo	HLAid-Sib-HLAid-Sib	19% (5–32%)		25% (1–49%)	
Cum. incidence chronic GVHD 5 yrs		40% (30–50%)		36% (21–51%)	
Death without cGVHD 5 yrs		45% (35–56%)		41% (25–56%)	
Chronic GVHD at 1 year	Other	34% (20–49%)	0.828	29% (11–47%)	0.802
	HLAid-Sib-HLAid-Sib	30% (18–42%)		21% (0–43%)	
Death without cGVHD 1 year	Other	25% (11–38%)	0.6	38% (18–57%)	0.942
	HLAid-Sib-HLAid-Sib	22% (11–33%)		29% (5–52%)	
Neutrophil engraftment Day +28		94% (CI 90–98%)		80% (69–92%)	
Death without engraftment Day +28		5% (CI 1–8%)		2% (0–6%)	

Bold values indicate statistical significance *P* < 0.05.

The number of transplants performed in each 5-year period for relapsed and graft failure patients is shown in Table 1B.

### Patients transplanted for relapsed MM

The median time from diagnosis to transplant was 16 (3–150) months for the first transplant and 69 (9–214) months for the second transplant.

In the relapse cohort of 159 patients, 86 had two HLA-identical sibling donor transplants and 70 had other donor types. Data was not available for three patients. Of the 86 classified as having had ‘two HLA-identical sibling donor’ transplants, information on whether they were transplanted using the same or a different donor was available in 42 of the 86 cases; a total of 35 (83%) had two consecutive transplants from the same HLA-identical sibling donor and seven (17%) has two different HLA-identical sibling donors. Of the 70 patients who had other donor types, information on whether they were transplanted using the same or a different donor was available in 59 of the 70 cases. A total of 51 (86%) had different donors for the two transplants. There were eight patients (14%) who were transplanted from the same donor of ‘other donor type’.

### Conditioning and GVHD prophylaxis

Conditioning regimens used in patients with relapsed disease were mostly fludarabine-based, included Fludarabine and Busulphan ±ATG (27%), Fludarabine and Melphalan ±ATG (22%), and Fludarabine and Treosulphan ±ATG (9%). Other regimens included Bulsulphan and Cyclophosphamide ±ATG (14%) and Busulphan and Melphalan ±ATG (8%).

GVHD prophylaxis consisted of cyclosporin-based approaches in 83% of patients in whom data was available, including cyclosporin and methotrexate (MTX) (42%) and cyclosporin and mycophenolate mofetil (MMF) (35%).

### GvHD

The rates of acute and chronic GVHD following the second transplant are shown in Table 1C.

When those patients with relapsed myeloma who had two consecutive HLA-identical sibling donor transplants (*n* = 86) were compared to all other donor combinations (*n* = 70), there was no difference in the rates of acute grade II-IV GvHD (HLAid-Sib/HLAid-Sib 34% (23–45%) versus other 25% (14–36%) (*P* = 0.2)).

When outcomes were then stratified based, firstly, on the presence or absence of GVHD following the first transplant and, secondly, on whether patients had had two HLA-identical sibling donor transplants as opposed to other donor combinations, there was a significantly higher incidence of acute grade II–IV GvHD in patients with prior GvHD who proceeded to a second HLA-identical sibling donor transplant (HLA-HLA: 50% (33–67%) vs. Other 22% (14–36%), *p* = 0.03).

### Overall survival

**Fig. 1:**
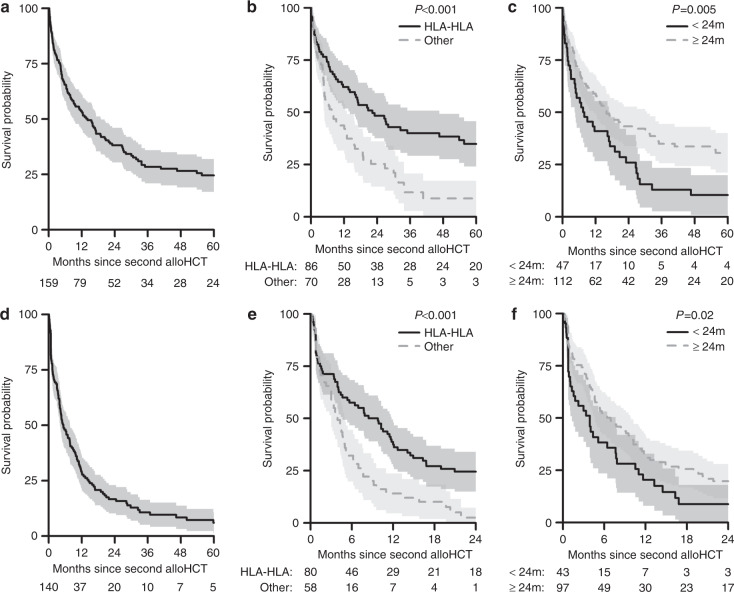
Overall and Progression-Free Survival following a second allogeneic transplant for relapsed myeloma and factors affecting these outcomes. **a** Overall survival of patients who underwent a second allo-HCT for relapsed myeloma. **b** Overall survival of patients who had two consecutive HLA-identical sibling donor transplants (HLA-HLA) versus other donor types. **c** Overall survival of patients who underwent a second allo-HCT for relapsed myeloma based on time between first and second allo-HCT (2-year cut-off). **d** Progression-free survival of patients who underwent a second allo-HCT for relapsed myeloma. **e** Progression-free survival of patients who had two consecutive HLA-identical sibling donor transplants (HLA-HLA) versus other donor types. **f** Progression-free survival of patients who underwent a second allo-HCT for relapsed myeloma based on time between first and second allo-HCT (2-year cut-off).

Univariate analysis of factors potentially affecting Overall Survival (OS) are shown in Table 2. OS was 38% (CI 30–46%) at 2 years post-transplant and 25% (CI 17–32%) at 5 years (Fig. 1a). There was no difference in OS between male and female patients. OS at 5 years was significantly superior when HLA-identical sibling donors were used for both transplants, compared to other donor types (35% (24–46%) vs. 9% (0–17%), *p* < 0.001) (Fig. 1b). Significantly inferior OS was also seen in those who proceeded to a second allo-HCT within 2 years when compared to those who were re-transplanted more than 2 years later (10% (1–20%) vs. 31% (21–40%) at 5 years, *P* = 0.005) (Fig. 1c).

**Table 2:** Univariate analysis for overall survival, progression-free survival, relapse and non-relapse mortality in patients transplanted for relapsed MM.

	OS				PFS				Relapse			NRM		
	*N*	2 year	5 year	*p*	*N*	2 year	5 year	*p*	2 year	5 year	*p*	2 year	5 year	*p*
Total														
All patients	159	38% (30–46%)	25% (17–32%)		140	17% (10–23%)	6% (1–11%)		68% (60–76%)	79% (71–86%)		15% (9–21%)	15% (9–21%)	
Patient gender														
Male	99	34% (25–44%)	20% (11–29%)	0.149	86	19% (10–28%)		0.679	67% (56–77%)		0.986	14% (7–22%)		0.679
Female	60	44% (31–57%)	32% (19–44%)		54	13% (4–23%)			70% (57–82%)			17% (7–27%)		
Donor type														
HLA-HLA	86	48% (38–59%)	35% (24–46%)	**<0.001**	80	24% (15–34%)		**<0.001**	64% (54–75%)		0.148	11% (4–18%)		0.091
Other	70	25% (14–36%)	9% (0–17%)		58	3% (0–7%)			75% (63–87%)			22% (11–34%)		
Interval between allos	HR = 0.94 (0.89–1)			0.0654		HR = 0.92 (0.86–0.98)		**0.0078**	HR = 0.89 (0.83–0.96)		**0.0021**	HR = 1.03 (0.9–1.17)		0.7126
<2 years	47	26% (13–39%)	10% (1–20%)	**0.005**	43	9% (0–18%)		**0.02**	77% (63–90%)		0.083	15% (4–25%)		0.896
≥2 years	112	43% (34–53%)	31% (21–40%)		97	20% (12–28%)			65% (55–74%)			16% (8–23%)		
Disease stage														
Low	60	42% (29–55%)	24% (11–37%)	0.747	53	18% (7–29%)		0.265	57% (44–71%)		**0.009**	25% (13–37%)		**0.03**
Advanced	85	33% (23–44%)	20% (10–29%)		78	12% (5–20%)			77% (68–87%)			11% (4–18%)		
Second HSCT year	HR = 1.02 (0.99–1.05)			0.2591	HR = 1.01 (0.98–1.03)			0.6997	HR = 1 (0.97–1.03)		0.8816	HR = 1.02 (0.95–1.09)		0.5503
1994–2008	81	35% (24–45%)	25% (15–34%)	0.377	71	18% (9–27%)		0.711	68% (57–78%)		0.489	14% (6–22%)		0.652
>2008	78	42% (30–53%)	22% (9–34%)		69	14% (5–23%)			69% (58–81%)			17% (8–26%)		
Age at 2nd Tx	HR = 1.09 (0.88–1.35)			0.4374	HR = 1.04 (0.84–1.28)			0.724	HR = 1.19 (0.93–1.50)		0.1618	HR = 0.58 (0.36–0.95)		**0.03**
<50	65	38% (26–51%)	29% (18–41%)	0.601	60	20% (10–30%)		0.665	56% (43–69%)		**0.021**	24% (13–35%)		**0.018**
50–70	94	38% (28–48%)	20% (10–30%)		80	14% (6–22%)			77% (67–87%)			9% (3–15%)		
Karnofsky score														
<90	33	30% (13–46%)	25% (8–41%)	0.212	28	12% (0–25%)		0.523	76% (60–93%)		0.142	11% (0–23%)		0.252
90–100	50	43% (29–57%)	20% (6–34%)		44	16% (5–28%)			60% (45–75%)			24% (11–36%)		
Stem cell source														
BM	15	46% (20–71%)	27% (2–52%)	0.966	14	7% (0–21%)		0.108	79% (57–100%)		0.196	14% (0–33%)		0.95
PB	141	38% (30–46%)	25% (17–33%)		125	18% (11–25%)			66% (58–75%)			16% (9–22%)		
Conditioning intensity														
Standard	60	37% (24–49%)	27% (15–40%)	0.702	52	15% (5–25%)		0.868	67% (54–80%)		0.98	18% (7–28%)		0.822
Reduced	73	43% (31–55%)	23% (12–34%)		66	19% (9–29%)			65% (53–77%)			16% (7–24%)		
MM														
IgG	77	44% (32–55%)	29% (18–40%)	0.282	69	24% (13–34%)		0.285	60% (48–72%)		0.385	16% (8–25%)		0.524
IgA	33	36% (20–53%)	15% (1–29%)		29	7% (0–16%)			72% (56–89%)			21% (6–35%)		
Light chain	36	32% (15–49%)	27% (11–44%)		32	12% (0–25%)			78% (63–93%)			10% (0–20%)		
EBMT risk score														
<5	21	57% (35–78%)	42% (18–66%)	0.086	20	34% (13–55%)		0.281	36% (15–58%)		0.057	30% (10–50%)		0.115
5	72	38% (27–50%)	18% (8–28%)		69	9% (2–16%)			75% (64–85%)			17% (8–25%)		
>5	51	27% (14–40%)	19% (7–31%)		41	15% (3–26%)			75% (62–89%)			10% (1–20%)		

Bold values indicate statistical significance *P *< 0.05.

### Progression-free survival

Univariate analysis of factors potentially affecting Progression-Free Survival (PFS) are shown in Table 2.

PFS was 17% (CI 10–23%) at 2 years post transplant and 6% (1–11%) at 5 years (Fig. 1d). There was no significant difference in PFS between male and female patients. PFS was significantly superior at 2 years when HLA-identical sibling donors were used for both transplants (24% (15–34%) vs. 3% (0–7%), *p* < 0.001) (Fig. 1e). Significantly inferior PFS at 24 months was seen in those who proceeded to a second allo-HCT within 2 years (9% (0–18%) vs. 20% (12–28%), *p* = 0.02) (Fig. 1f).

### Relapse incidence/non-relapse mortality

**Fig. 2:**
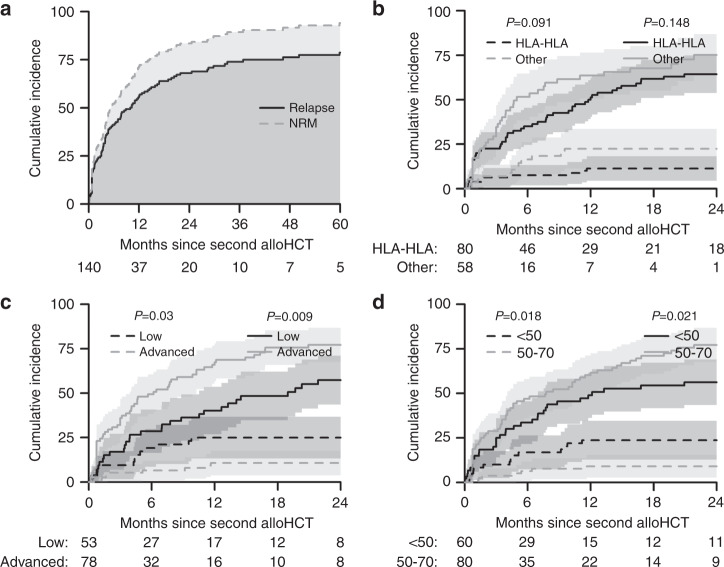
Relapse incidence and NRM rates following a second allogeneic transplant for relapsed myeloma and factors affecting these outcomes. **a** Cumulative incidence of relapse (solid line) and non-relapse mortality (dashed line) over 5 years in patients whose second allo-HCT was for relapsed myeloma. **b** Cumulative incidence of relapse (solid line) and non-relapse mortality (dashed line) over 5 years based on the type of stem cell donor (HLA-HLA) in patients whose second allo-HCT was for relapsed myeloma. **c** Cumulative incidence of relapse (solid line) and non-relapse mortality (dashed line) over 5 years based on Disease Stage (low or advanced) in patients whose second allo-HCT was for relapsed myeloma. **d** Cumulative incidence of relapse (solid line) and non-relapse mortality (dashed line) over 5 years based on age of transplant recipient in patients whose second allo-HCT was for relapsed myeloma.

Relapse Incidence (RI) and NRM rates in patients transplanted for relapsed MM are shown in Table 2. The RI and NRM at 2 and 5 years are shown in Fig. 2a.

Disease status affected RI and NRM. The incidence of relapse was significantly higher at 2 years (77% (68–87%) vs. 57% (44–71%)) (*p* = 0.009) in patients with advanced disease (Fig. 2c). Conversely, the cumulative NRM was significantly higher at 2 years (25% (13–37%) vs. 11% (4–18%) (*p* = 0.03)) in patients with low disease burden (Fig. 2c). Older age at second allo-HCT (50–70 vs. <50 years) was associated with a higher RI at 2 years (77% (67–87%) vs. 56% (43–69%), *p* = 0.021) and the cumulative NRM was significantly higher in younger individuals at 2 years (24% (13–35%) vs. 9% (3–15%)) (*p* = 0.018) (Fig. 2d).

### Multivariate analysis

Multivariate analysis results are shown in Table 3.

**Table 3:** Multivariate analysis in relapse cohort for overall survival, progression-free survival, and relapse.

	Overall survival				Progression-free survival				Relapse			
Risk Factor	*N*	N-event	HR (95% CI)	*p*	*N*	N-event	HR (95% CI)	*p*	*N*	N-event	HR (95% CI)	*p*
Total					99	87			99	70		
Patient gender												
Male	65	49			59	49			59	39		
Female	45	36	0.66 (0.41–1.08)	0.1021	40	38	1.13 (0.71–1.79)	0.6049	40	31	1.21 (0.72–2.01)	0.4689
Donor type												
Other	53	43			44	38			44	28		
HLA-HLA	57	42	0.4 (0.24–0.67)	**<0.001**	55	49	0.53 (0.31–0.91)	**0.0219**	55	42	0.7 (0.39–1.27)	0.2373
Disease stage												
Low	51	39			45	38			45	26		
Advanced	59	46	0.94 (0.58–1.53)	0.8145	54	49	1.28 (0.79–2.07)	0.3216	54	44	1.85 (1.05–3.24)	**0.0327**
Conditioning												
Standard	52	42			45	40			45	32		
Reduced	58	43	0.85 (0.55–1.32)	0.4654	54	47	1.03 (0.66–1.59)	0.9093	54	38	1.09 (0.67–1.79)	0.7297
Previous GvHD												
No previous GvHD	52	43			46	44			46	37		
Any previous GvHD	58	42	0.68 (0.42–1.1)	0.1165	53	43	0.71 (0.44–1.15)	0.1614	53	33	0.66 (0.38–1.14)	0.1338
Age (decades)	110	85	1.22 (0.89–1.68)	0.2055	99	87	1.09 (0.79–1.51)	0.5831	99	70	1.28 (0.88–1.86)	0.2013
Interval to T x 2	110	85	0.98 (0.9–1.07)	0.6776	99	87	0.94 (0.86–1.03)	0.1945	99	70	0.88 (0.79–0.98)	**0.0186**

Bold values indicate statistical significance *p* < 0.05.

When adjusting for the indicated covariates, patients who received both their first and second transplants from HLA-identical siblings had better OS (HR = 0.4 (0.24–0.67), *p* < 0.001) and PFS (HR = 0.53 (0.31–0.91)) than other combinations. The risk of relapse was higher in those with more advanced disease (1.85 (1.05–3.24)), *p* = 0.033) and when there was a shorter interval between transplants (0.88 (0.79–0.98), *p* = 0.019).

### Patients transplanted for graft failure

#### Conditioning and GVHD prophylaxis

Conditioning regimens used in patients with graft failure were mostly fludarabine-based, included Fludarabine and Busulphan ±ATG (24%), Fludarabine and Melphalan ±ATG (22%), Fludarabine and Cyclophosphamide ±ATG (14%) and single agent Fludarabine (14%). A total of 14% of patients in whom data was available were conditioned with Cyclophosphamide ±ATG or Alemtuzumab.

GVHD prophylaxis consisted of cyclosporin-based approaches in 93% of patients in whom data was available, including cyclosporin and methotrexate (MTX) (28%), cyclosporin and MMF (26%) and single agent cyclosporin (30%).

#### Graft-versus-host disease

In the first 100 days following the second allo-HCT, the rate of grade II acute GvHD was 12% (3–22%)), grade III–IV acute GVHD 15% (5–25%)) and death without GvHD 17% (6–27%)). The 2-year cumulative incidence of chronic GvHD was 36% (21–51%)) and death without chronic GvHD 41% (25–56%)).

#### Overall survival and progression-free survival

**Fig. 3:**
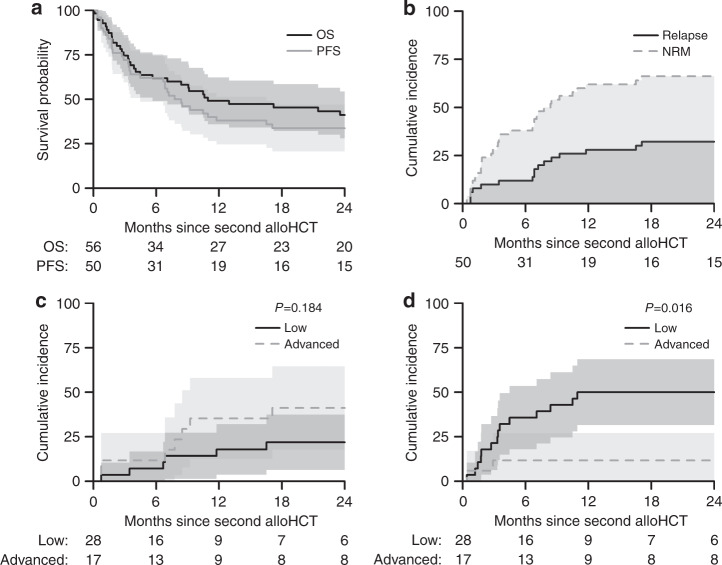
Overall and Progression-Free Survival in patients with myeloma following a second allogeneic transplant for graft failure and factors affecting these outcomes. **a** Two-year overall survival and progression-free survival of MM patients who underwent a second allo-HCT for graft failure. **b** Cumulative incidence of relapse (solid line) and non-relapse mortality (dashed line) over 2 years in MM patients whose second allo-HCT was for graft failure. **c** Two-year cumulative incidence of relapse based on disease stage (low or advanced) in MM patients whose second allo-HCT was for graft failure. **d** Two-Year cumulative incidence of non-relapse mortality based on disease stage (low or advanced) in MM patients whose second allo-HCT was for graft failure.

OS was 41% (CI 28–54%) and PFS 34% (CI 21–47%) at 2 years post-transplant (Fig. 3a). Univariate analysis of factors potentially affecting OS and PFS is shown in Table 4. None of these factors affected outcomes. The causes of death are shown in Table 5.

**Table 4:** Univariate analyses for Overall Survival, Progression-Free Survival, Relapse and NRM in patients transplanted for graft failure.

	Overall survival			Progression-free survival			Relapse		Non-relapse mortality	
	*N*	2 years	*p* value	*N*	2 years	*p* value	2 years	*p* value	2 years	*p* value
All	56	41% (28–54%)		50	34% (21–47%)		32% (19–45%)		34% (21–47%)	
Patient gender										
Male	24	41% (21–61%)	0.771	22	31% (12–51%)	0.925	28% (9–47%)	0.538	41% (20–61%)	0.489
Female	32	41% (24–59%)		28	36% (18–53%)		36% (18–53%)		29% (12–45%)	
Donor type										
HLAid-Sib-HLAid-Sib	20	32% (11–52%)	0.699	18	28% (7–48%)	0.699	39% (16–61%)	0.429	33% (12–55%)	0.726
Other	34	47% (30–64%)		31	35% (18–52%)		29% (13–45%)		35% (19–52%)	
Interval between allos										
<1 year	44	37% (22–51%)	0.266	38	37% (22–52%)	0.585	26% (12–40%)	0.147	37% (22–52%)	0.567
≥1 year	12	58% (30–86%)		12	25% (1–49%)		50% (22–78%)		25% (1–49%)	
Disease status										
Low	29	37% (19–55%)	0.147	28	28% (11–45%)	0.21	22% (6–37%)	0.184	**50%** (**31–69%)**	**0.016**
Advanced	19	61% (39–84%)		17	47% (23–71%)		41% (18–65%)		**12%** (**0–27%)**	
Year at second allo										
1994–2008	28	33% (15–51%)	0.341	23	39% (19–59%)	0.458	22% (5–39%)	0.191	39% (19–59%)	0.65
After 2008	28	49% (30–68%)		27	30% (12–47%)		41% (22–59%)		30% (12–47%)	
Age at second allo										
<50 years	29	44% (26–63%)	0.868	27	37% (19–55%)	0.873	30% (12–47%)	0.816	33% (16–51%)	0.942
50–70 years	27	38% (19–57%)		23	30% (12–49%)		35% (15–54%)		35% (15–54%)	
Karnofsky score										
<90	13	62% (35–88%)	0.345	13	38% (12–65%)	0.488	38% (12–65%)	0.684	23% (0–46%)	0.269
90–100	16	42% (16–67%)		16	25% (4–46%)		31% (9–54%)		44% (19–68%)	
Stem cell source										
BM	6	50% (10–90%)	0.582	6	50% (10–90%)	0.349	17% (0–46%)	0.367	33% (0–71%)	0.904
PB	49	39% (25–53%)		43	30% (16–44%)		35% (21–50%)		35% (21–49%)	
Conditioning intensity										
Standard	15	39% (14–64%)	0.823	12	33% (7–60%)	0.845	42% (14–70%)	0.754	25% (1–49%)	0.711
Reduced	33	46% (29–64%)		30	36% (19–54%)		34% (17–51%)		30% (14–46%)	
MM										
IgG	25	44% (25–63%)	0.834	22	40% (20–61%)	0.629	28% (9–47%)	0.637	32% (12–51%)	0.879
IgA	13	31% (6–56%)		12	25% (1–49%)		42% (14–70%)		33% (7–60%)	
Light chain	18	46% (22–70%)		16	31% (9–54%)		31% (9–54%)		38% (14–61%)	
EBMT risk score										
<5	11	34% (5–63%)	0.682	11	34% (5–63%)	0.867	20% (0–46%)	0.527	45% (16–75%)	0.826
5	22	43% (22–64%)		19	37% (15–59%)		32% (11–52%)		32% (11–52%)	
>5	15	52% (27–78%)		15	27% (4–49%)		40% (15–65%)		33% (9–57%)	

Bold values indicate statistical significance *p* < 0.05.

**Table 5:** Causes of death within the follow-up period: 5 years for relapse, 2 years for graft failure.

Causes of death	Relapsed myeloma			Graft failure		
	Missing	Frequency	Percent	Missing	Frequency	Percent
Relapse/disease progression	3	59	55.1	1	7	22.6
Secondary malignancy/PTLD		2	1.9			
GVHD		12	11.2		4	12.9
Infection		21	19.6		10	32.3
Organ damage/failure		2	1.9		3	9.7
Toxicity		1	0.9		2	6.5
HSCT-related death		5	4.7		2	6.5
Other		5	4.7		3	9.7
Total		110			32	

#### Relapse/non-relapse mortality

Two-year relapse and NRM rates in patients transplanted for graft failure are shown in Table 4 and Fig. 3b. After 2 years, the cumulative RI was 32% (19–45%)) and NRM 34% (21–47%). The 2-year incidence of relapse based on disease stage is shown in Fig. 3c.

In addition, NRM at 2 years was higher in those with a low disease burden when compared to those with an advanced disease burden ((50% (31–69%) vs. 12% (0–27%) (*p* = 0.016) (Fig. 3d).

## Discussion

This is the first registry report of outcomes following second allo-HCT in patients with myeloma. In this analysis, the indications for re-transplantation fell into two groups: relapse and graft failure. In the relapse group, OS was 38% at 2 years and 25% at 5 years. This is marginally better than the aggregate 20% 5-year OS rate reported by Ruutu and colleagues for all 2632 second allogeneic transplants performed in EBMT centres for disease relapse between 1994 and 2009 [13]. In that EBMT registry study, the use of a HLA-identical sibling donor for the second transplant was found to be a favourable predictive factor for OS with a 5-year OS probability of 22% as opposed to 17% using either other related or unrelated donors (*P* = 0.012). However, whether the same or a new donor was used for the second transplant appeared to make no difference.

In order to more clearly assess these overlapping and potentially confounding categories (HLA-matched versus non-HLA-matched, same donor versus change of donor), we focused on the outcomes of patients who had received both their first and second transplants from HLA-identical sibling donors. Based on the available registry data, over four-fifths of these patients had the same HLA-identical sibling donor for the two transplants.

In our cohort, patients who had two consecutive HLA-identical sibling donor transplants had better PFS at 2 years (24% vs. 3%, *p* = <0.001) and OS at 5 years (35% vs. 9%, *P* < 0.001). This was confirmed on multivariate analysis with patients receiving both their first and second transplants from HLA-identical sibling donors having better OS (HR = 0.4 (0.2–0.7), *p* < 0.001) and PFS (0.5 (0.3–0.9), *p* = 0.022). Conversely, there were no survivors among the eight patients who had the same donor of any other category (all donor types except for HLA-identical sibling) for both transplants. The positive effect of keeping the same donor, therefore, appears to depend on the specific donor type rather than the individual stem cell donor. One interpretation of these results might be that any additional alloimmune effect conferred by the use of an unrelated donor in transplantation for myeloma is outweighed by greater NRM. In those relapsed patients who had developed acute grade II-IV GVHD following their first allo-HCT, there was a significantly higher incidence of acute grade II–IV GvHD when the same HLA identical sibling donor was used again (50% vs. 22%, *p* = 0.03).

Among patients who have a second allo-HCT for relapsed disease, those with shorter remissions following their first transplant have consistently been reported to have poorer outcomes [5, 6, 12, 18, 19]. This likely reflects both the earlier relapse of biologically more aggressive disease and the cumulative effect of treatment-related toxicity. The median interval between transplants in our relapse cohort was over 3 years. Those patients with relapsed disease who proceeded to a second allo-HCT within 2 years had a shorter PFS at 2 years (9% vs. 20%, *p* = 0.02) as well as a poorer OS at 5 years (10% vs. 31%, *p* = 0.005) when compared to those transplanted more than 2 years later. Also consistent with past reports, the relapse incidence at 2 years was higher (77% vs. 57%, *p* = 0.009) in patients whose disease was in less than a partial remission pre-transplant, when compared to those with a lower disease burden prior to the second transplant [6].

The use of either myeloablative conditioning or RIC prior to a first allo-HCT in patients with myeloma was recently reported to result in similar outcomes [20, 21]. Most of the acute leukemia literature favours the use of RIC in second allogeneic transplants due to lower NRM. In our relapse cohort, 59% of the first and 55% of the second transplants were performed following RIC. In the graft failure cohort, the equivalent percentages were 79% and 69%, respectively. Relapse was more common in older patients (50–70 years) though this did not reflect the intensity of conditioning as there was no difference in the frequency of use of reduced intensity conditioning approaches between the younger and older cohorts.

The causes of death differed between the relapse and graft failure cohorts. A total of 55% of the relapse group died of ‘relapse/disease progression’ as opposed to 20% of infection and 3% of organ damage/failure and toxicity. In the graft failure group, 23% died of subsequent relapse/disease progression, 32% of infection and 16% of organ damage/failure and toxicity. The shorter time between transplants and the prolonged neutropenia in the setting of graft failure are likely factors in this increased toxicity.

In the graft failure group, OS was 41% at 2 years. The NRM rate at 2 years in those patients whose myeloma was in a partial remission or better pre-transplant, was 50.2% (31–69%) compared to 12% (0–27%) in patients with a higher disease burden (*p* = 0.016). No other variable was found to significantly affect outcomes though this may partially reflect the relatively small patient cohort.

Although second allo-HCT is feasible in myeloma patients, OS outcomes remain poor. There is a need for strategies to achieve deeper remissions prior to any second allo-HCT. A focus on novel maintenance strategies to reduce the risk of relapse is also required [22, 23]. In a CIBMTR comparison of post-relapse OS after autologous/allogeneic (auto/allo) as opposed to tandem autologous (auto/auto) transplantation between 2000 and 2010, there was superior OS following relapse in auto/allo HCT recipients compared with auto/auto HCT recipients [24]. The authors contended that this reflected a better response to salvage with agents such as immunomodulatory drugs in the context of donor-derived immunity. This detailed information is unfortunately not available to allow us to analyse the relevance of such factors in our cohort. Nonetheless, small molecule inhibitors and novel immunotherapies have the potential to change the kinetics of disease relapse, thereby facilitate an emerging alloimmune graft-versus-malignancy response post transplant.

Myeloma patients who relapse post-transplant often retain full donor chimerism. In this setting, Novak and colleagues reported on second “allogeneic” transplants using CD34+ selected donor cells without immunosuppression followed by DLI and/or maintenance therapy as a means of achieving disease control [17]. Myeloablative conditioning was well tolerated with no NRM and no GVHD. OS was 100% at 1 year and 69% at 2 years though PFS was only 13% at 2 years.

This may represent another salvage treatment option in selected patients.

There are some weaknesses in our study. The disease staging system, an EBMT remission-based classification, is not specific to myeloma and it is surprising that there is no difference in OS or PFS between those with ‘Low’ as opposed to ‘Advanced’ disease. It is possible that the very high rates of post-transplant relapse––overall PFS was 6% (1–11%) at 5 years in patients transplanted for relapsed myeloma––may have masked any PFS difference. In addition, OS will have been affected by subsequent salvage treatments. However, there is a signal of their ‘Advanced’ disease status is that these patients have a significantly higher relapse rate on both univariate and multivariate analysis.

In summary, one quarter of myeloma patients remained alive 5 years following a second allogeneic haematopoietic cell transplant with similar outcomes seen following disease relapse and graft failure. The best outcomes (35% OS at 5 years) were seen in those who had two consecutive HLA-identical sibling donor transplants.

## Contributors

Yves Beguin, University of Liege, Liege, Belgium; Jean Henri Bourhis, Gustave Roussy Cancer Campus, Villejuif, France; Peter Dreger, University of Heidelberg, Heidelberg, Germany; Franca Fagioli, Onco-Ematologia Pediatrica, Torino, Italy; Jürgen Finke, University of Freiburg, Freiburg, Germany; Hélène Labussière-Wallet, Centre Hospitalier Lyon Sud, Lyon, France; Matjaz Sever, University Med. Center, Ljubljana, Slovenia; Gwendolyn Van Gorkom, University Hospital Maastricht, Maastricht, Netherlands; Jane Apperley, Imperial College, London, UK; Wolfgang Bethge, Universitaet Tuebingen, Tuebingen, Germany; Didier Blaise, Programme de Transplantation&Therapie Cellulaire, Marseille, France; Francesca Bonifazi, Bologna University, S.Orsola-Malpighi Hospital, Bologna, Italy; Prof. J.J. Cornelissen, Erasmus MC Cancer Institute, Rotterdam, Netherlands; Ahmet Elmaagacli, Asklepios Klinik St. Georg, Hamburg, Germany; John Gribben, St. Bartholomew’s and The Royal London NHS Trust, London, UK; Maija Itäla-Remes, Turku University Hospital, Turku, Finland; Yener Koc, Medicana International Hospital Istanbul, Istanbul, Turkey; Xavier Leleu, Hopital La Miletrie, Poitiers, France; Giuseppe Marotta, U.O.S.A Centro Trapianti e Terapia Cellulare, Siena, Italy; Francesco Onida, Fondazione IRCCS - Ca’ Granda, Milano, Italy; Kim Orchard, Southampton General Hospital, Southampton, UK; Dominik Selleslag, A.Z. Sint-Jan, Brugge, Belgium; Lorenz Thurner, University of Saarland, Homburg, Germany; Dominik Wolf, University Hospital Innsbruck, Innsbruck, Austria; Gerald. G. Wulf, Universitaetsklinikum Goettingen, Goettingen, Germany; Ibrahim Yakoub-Agha, CHU de Lille, Lille, France; Adrián Alegre Amor, Hospital de la Princesa, Madrid, Spain; Achilles Anagnostopoulos, George Papanicolaou General Hospital, Thessaloniki, Greece; Grzegorz Basak, Central Clinical Hospital, Warsaw, Poland; Jacques-Olivier Bay, CHU ESTAING, Clermont_Ferr, France; Martin Bornhäuser, Universitaetsklinikum Dresden, Dresden, Germany; Claude Eric Bulabois, CHU Grenoble Alpes - Université Grenoble Alpes, Grenoble, France; Dolores Caballero, Hospital Clínico, Salamanca, Spain; Thomas Cluzeau, CHU Nice - Hôpital de l’ARCHET I, Nice, France; Matthew Collin, Adult HSCT unit, Newcastle_Tyne, UK; Paolo Corradini, University of Milano, Milano, Italy; Charles Craddock, University Hospital Birmingham NHSTrust, Birmingham, UK; Eric Deconinck, Hopital Jean Minjoz, Besancon, France; J.L. Diez-Martin, Hospital Gregorio Marañón, Madrid, Spain; Matthias Edinger, University Regensburg, Regensburg, Germany; Tobias Gedde-Dahl, Oslo University Hospital, Rikshospitalet, Oslo, Norway; Cecilia Isaksson, Umea University Hospital, Umea, Sweden; Wu Ka Lung, ZNA, Antwerp, Belgium; Tessa Kerre, Ghent University Hospital, Gent, Belgium; Anjum Khan, Yorkshire Blood & Marrow Transplant Programme, Leeds, UK; Guido Kobbe, Heinrich Heine Universitaet, Duesseldorf, Germany; Giorgio La Nasa, Centro Trapianti Unico Di CSE Adulti e Pediatrico A. O Brotzu, Cagliari, Italy; Andrzej Lange, DCTK, Wroclaw, Poland; Murray Martin, Leicester Royal Infirmary, Leicester, UK; Ellen Meijer, VU University Medical Center, Amsterdam, Netherlands; Mohamad Mohty, Hopital Saint Antoine, Paris, France; Nicola Mordini, Az. Ospedaliera S. Croce e Carle, Cuneo, Italy; Arnon Nagler, Chaim Sheba Medical Center, Tel_Hashomer, Israel; Zubeyde Nur Ozkurt, Gazi University Faculty of Medicine, Ankara, Turkey; Amit Patel, Clatterbridge Cancer Centre - Liverpool, Royal Liverpool University Hospital, Liverpool, UK; Victoria Potter, Kings College Hospital, London, UK; Ron Ram, Tel Aviv Sourasky Medical Center, Tel_Aviv, Israel; Alessandro Rambaldi, ASST Papa Giovanni XXIII, Bergamo, Italy; Christian Reinhardt, University Hospital, Essen, Germany; Riccardo Saccardi, Azienda Ospedaliera Universitaria Careggi, Firenze, Italy; Jaime Sanz, University Hospital La Fe, Valencia, Spain; Urs Schanz, University Hospital, Zurich, Switzerland; Christof Scheid, University of Cologne, Cologne, Germany; Kerstin Schäfer-Eckart, Klinikum Nuernberg, Nuernberg, Germany; Rosanna Scimè, U.O.D Trapianti di midollo osseo, Palermo, Italy; Henrik Sengeloev, Bone Marrow Transplant Unit L 4043, Copenhagen, Denmark; Simona Sica, Universita Cattolica S. Cuore, Rome, Italy; Gerard Socié, Hopital St. Louis, Paris, France; Abdelghani Tbakhi, King Hussein Cancer Centre, Amman, Jordan; Herve Tilly, Centre Henri Becquerel, Rouen, France; Thomas Valerius, University Medical Center Schleswig-Holstein, Campus Kiel, Kiel, Germany; Andrea Velardi, Sezione di Ematologia, Perugia, Italy; Jan Vydra, Institute of Hematology and Blood Transfusion, Prague, Czech Rep; Eva Maria Wagner-Drouet, University Medical Center Mainz, Mainz, Germany; Jan Walewski, Maria Sklodowska-Curie Institute - Oncology Centre, Warsaw, Poland; Pavel Zák, Charles University Hospital, Hradec_Kralove, Czech Rep.
